# Co-Responses of Soil Organic Carbon Pool and Biogeochemistry to Different Long-Term Fertilization Practices in Paddy Fields

**DOI:** 10.3390/plants11233195

**Published:** 2022-11-22

**Authors:** Young-Nam Kim, Ji-Hyun Lee, Han-Ryul Seo, Jeong-Woo Kim, Young-Sang Cho, Danbi Lee, Bo-Hyun Kim, Jung-Hwan Yoon, Hyeonji Choe, Yong Bok Lee, Kye-Hoon Kim

**Affiliations:** 1 Department of Environmental Horticulture, University of Seoul, Seoul 02504, Republic of Korea; 2 Division of Applied Life Science (BK21), Gyeongsang National University, Jinju 52828, Republic of Korea; 3 Institute of Agriculture and Life Science (IALS), Gyeongsang National University, Jinju 52828, Republic of Korea; 4 Department of Agricultural Environment, National Institute of Agricultural Science, Wanju 55365, Republic of Korea; 5 Kangwon Institute of Inclusive Technology, Kangwon National University, Chuncheon 24341, Republic of Korea

**Keywords:** rice field, organic amendments, liable carbon pool, carbon sequestration, soil fertility, methane emission, sustainable land management

## Abstract

Long-term application of soil organic amendments (SOA) can improve the formation of soil organic carbon (SOC) pool as well as soil fertility and health of paddy lands. However, the effects of SOA may vary with the input amount and its characteristics. In this work, a descriptive field research was conducted during one cropping season to investigate the responses of various SOC fractions to different long-term fertilization practices in rice fields and their relationships with soil biogeochemical properties and the emission of greenhouse gases (GHG). The field sites included two conventional paddies applied with chemical fertilizer (CF) or CF + rice straw (RS) and six organic agriculture paddies applied with oilseed cake manure (OCM) + wheat straw (WS), cow manure (CM) + WS, or CM + RS. The two paddy soils treated with CM + RS had significantly higher concentrations of recalcitrant to labile C forms, such as loss-on-ignition C (LOIC; 56–73 g kg^−1^), Walkley–Black C (WBC; 20–25 g kg^−1^), permanganate oxidizable C (POXC; 835–853 mg kg^−1^), and microbial biomass carbon (MBC; 133–141 mg kg^−1^), than soils treated with other SOA. Likewise, long-term application of CM + RS seemed to be the best for regulating soil fertility parameters, such as ammonium (11–141 mg kg^−1^); phosphate (61–106 mg kg^−1^); and soluble Ca, K, and Mg (7–10, 0.5–1.2, and 1.9–3.8 mg kg^−1^, respectively), although the results varied with the location and soil properties of rice fields. Additionally, the two paddy sites had the largest cumulative methane emission (754–762 kg ha^−1^), seemingly attributed to increased microbial biomass and labile C fractions. The significant correlations of most SOC fractions with soil microbial biomass, trophic factors, and methane emissions were confirmed with multivariate data analysis. It was also possible to infer that long-term SOA application, especially with CM + RS, enhanced interaction in belowground paddy fields, contributing to soil fertility and rice production sustainability. Based on our findings, we suggest the need for analysis of various types of SOC fractions to efficiently manage soil fertility and quality of paddy fields, C sequestration, and GHG emissions.

## 1. Introduction

Agricultural soil fertility and quality can be constantly improved by sustainable soil management practices, which contributes to enhancing crop production and quality. Since about the 20th century BC, various organic byproducts (e.g., compost, crop residues, livestock manures, etc.) have been amended into arable lands as fertilization sources. Chemical fertilizers have been widely introduced and used as a booster of crop production over the past 100 years as the global population boomed. Thus, it is likely to meet the growing demands for food production stability to some extent. However, severe degradation of the soil quality (e.g., the decline in soil organic matter (SOM), nutrient depletion, soil acidification, decrease in microbial diversity, etc.) has been caused by the constant and excessive application of chemicals, thereby affecting crop production sustainability [1,2]. Hence, to compensate for the shortcomings caused by the abuse of chemical fertilizers, widespread attempts are currently being made to improve the status of SOM, which is strongly linked to soil function sustainability, in agricultural lands globally [2,3,4]. In addition, other appropriate soil management practices, such as tillage reduction, cover cropping, and crop rotations, have been conducted in modern farmlands to increase the efficiency of sustainable soil management [5,6].

In terrestrial ecosystems, SOM is a primary factor for regulating and sustaining soil functions, such as the dynamics of water and nutrients, the provision of food sources to soil biota, and the remediation of soil contamination [2,7,8]. Therefore, SOM can be recognized as a critical contributor to the improvement of crop performance. Various soil organic amendments (SOA), including composts, manures, biochar, vermicompost, etc., are used in different arable lands, such as paddies, to increase SOM. As reported in many studies, the main benefit of SOA application is undoubtedly an increase in soil organic carbon (SOC) stock and improvements in overall soil characteristics, e.g., aggregate stability, water infiltration and retention, nutrient cycling, biodiversity conservation, etc. [9,10,11]. Furthermore, according to a meta-analysis of the global soil dataset conducted by Luo et al. [12], rather than inorganic fertilization alone, better results could be obtained in multiple soil systems using fertilization practices accompanied by SOA, such as improvement of soil structure and fertility and maintenance of soil health. Luo et al. [12] also comprehensively suggested that crop productivity could be increased by improved microbe-mediated soil ecosystem functions and services, such as increase in nitrogen and carbon use efficiency, through SOA application. However, the benefits of SOA application may vary significantly with geographic and climatic differences and farming systems [12,13]. 

The SOC stock in agricultural land is determined by the balance between C inputs and degradability. It can be maintained at a high equilibrium level by constantly adding SOA, including compost, manure, and crop residues [14]. However, depending on the nature of SOA (i.e., stability and decomposition rate, which are primarily measured by soil biota), the effects of SOA input on the SOC stock and its structural components can vary significantly. Therefore, the overall soil biogeochemical status in agroecosystems is affected. Practically, total soil organic C is quantified through the loss-on-ignition (LOI) and Walkley–Black (WB) wet oxidation methods. These methods have been used globally to evaluate the fertility and quality of agricultural soils for effective land management. The values of LOI-based C (LOIC) and WB-based C (WBC) are in recalcitrant form. However, their inadequacies as a soil quality assessment indicator have occasionally been observed through the analysis of their poor relationships with significant soil variables associated with nutrient status and microbial biomass and activity [2,11]. Meanwhile, labile soil organic C (LSOC) better facilitates microbiota and the nutrient cycle than C in recalcitrant form [15]. LSOC is more commonly recognized as a critical SOC fraction in farmlands to mediate the essential functions of soil and C sequestration [11,16,17]. Additionally, the high sensitivity of LSOC fractions, such as particulate organic C (POC), permanganate oxidizable C (POXC), potentially mineralizable C (PMC), microbial biomass C (MBC), dissolved organic C (DOC), etc., in response to changes in soil management practices or environmental factors has been proven in many studies [2,18,19,20,21]. This indicates their high utilization value as a soil quality indicator for predicting the productivity of agricultural soils [11,14,22]. 

In modern rice farming, organic fertilization alone or in combination with chemical fertilizers is becoming more popular than chemical fertilization alone to achieve better SOM status, soil nutrition balance, and microbial community structure and thus influence rice yield [22,23]. Additionally, organic agricultural practices are attracting attention in relation to eco-friendly farming, soil conservation, and economic feasibility [2]. Many previous experimental studies have demonstrated that the long-term application of SOA in paddy fields would bring benefits to sustainable rice production [24,25,26]. These attempts have been applied to many paddy fields in South Korea over the past decades. Policy and scientific efforts are underway to improve SOC stock in paddy fields. In addition, organic farming practices are encouraged to maximize spin-offs, such as increased C sequestration, improved soil fertility and ecological services, and reduced greenhouse gas (GHG) emissions. However, there have been no studies confirming whether these theoretical and experimental results are well represented in paddy fields in situ. Hence, this study was devised as a field descriptive research to investigate the effects of differences in land management in actual paddy fields by examining the responses of SOC fractions to different long-term fertilization practices and their relationships with soil biogeochemical properties and GHGs. For this, we selected various paddy fields that have been performing different agricultural practices for a long time in the same township (within a radius of 1 km) in Chungcheongnam-do, South Korea. We compared and interpreted the differences in soil carbon pool structure and biogeochemical properties according to fertilization practices. Through this field research, we expected to determine the most efficient soil management practice for paddy lands by analyzing the interaction between several types of SOC fractions and soil parameters.

## 2. Materials and Methods

### 2.1. Study Area and Land Management Practice

A total of eight paddy fields in Geumpyeong-ri, Hongdong-myeon, Hongseong-gun, and Chungcheongnam-do, South Korea, were selected for this study (Figure S1). These farmlands have more than 10 years of constant history of using land management practices to produce the early-maturing rice plant *Oryza sativa* cv. Jomyeong, which is cultivated from late June to early October. The fertilization practice methods for each paddy field are described in Table 1. Among the fields, two were conventional farmlands with additions of NPK (N:P_2_O_5_:K_2_O = 63:30:21; kg ha^−1^) alone and in combination with rice straw. The remaining paddies have been constantly practicing organic agriculture by applying organic amendments in different combinations, including cow manure (CM; 30 t ha^−1^), oilseed cake manure (OCM; 2 t ha^−1^), rice straw (RS; 100% turnover of the previous year’s yield), or wheat straw (WS; 100% turnover of the winter fallow’s yield). The properties of these organic amendments are shown in Table S1. The basal fertilizers were applied to the fields about 2−3 weeks before rice transplanting, and the additional inorganic fertilizer (N:K_2_O = 63:9, kg ha^−1^) for C1 and C2 and manure compost (containing 9% N, 200 kg ha^−1^) for O1 to O6 were added as topdressing fertilizers at the spike stage. The rice planting was conducted consistently in all paddies with a planting density of 25 seedlings m^−2^. In winter fallow, wheat seeds (75 kg ha^−1^) were sown in all fields in the middle of October and cultivated the following March. Primary tillage was performed after rice harvest, followed by secondary tillage conducted four times to control weeds and prepare the seedling bed before planting for the next year. 

### 2.2. Soil Collection

The topsoil (0–20 cm depth) of each field was collected one week after the rice harvest. Approximately 3 kg of paddy soil was sampled in triplicate and moved to a Soil Environment Laboratory at the University of Seoul to analyze the physical, biological, and chemical properties of the soil. Of the collected samples, 2 kg of soil was air-dried and stored in a container before analyzing the soil properties. Meanwhile, the remaining soil was kept in a refrigerator at 4 °C before soil microbial biomass carbon (MBC) analysis. Additionally, core sampling was conducted at each field using stainless steel soil core samplers of 100 cm^3^ volume to evaluate the soil bulk density (BD).

### 2.3. Analytical

#### 2.3.1. SOC Fractions

Four types of organic C fractions in soil samples were determined according to their degradability in this study, namely, loss-on-ignition C (LOIC), Walkley–Black C (WBC), permanganate oxidizable C (POXC), and potentially mineralizable C (PMC), which correspond to recalcitrant, slightly labile, moderate labile, and readily degradable SOC forms, respectively [2]. The predried soil at 105 °C was analyzed following the LOI method at 550 °C in a muffle furnace to determine the LOIC [27]. WBC in the soil sample was determined with the Walkley–Black chromic acid wet oxidation–titration method [28]. POXC, referred to as active C, in the soil was analyzed through the MnO_4_^−^ oxidation process described by Kim et al. [2]. The absorbance of the sample was measured at 550 nm using a microplate reader (Epoch, BioTek, Winooski, VT, USA). The POXC concentration was calculated using the formula specified by Hurisso et al. [17]. For PMC assessment, air-dried soil was rewetted by adjusting to a 50% water-filled pore space and incubated in a closed container for a day at 25 °C. The concentration of CO_2_ flush was determined using the GT5000 Terra portable gas analyzer (Gasmet Technologies, Vantaa, Finland) [2]. Furthermore, MBC in the soil sample was analyzed by a fumigation process with ethanol-free CHCl_3_ as outlined by Vance et al. [29]. Following extraction with 0.5 M K_2_SO_4_, the concentration of organic C in the extracts was determined using a TOC-L analyzer (Shimadzu, Kyoto, Japan).

#### 2.3.2. Soil Physical and Chemical Parameters

The soil BD was estimated through the core method [30] using the total soil volume of the cylinder (100 cm^3^). After oven drying at 105 °C for 3 d, the mass of the dried sample was recorded. The BD value was calculated using the formula described by Kim et al. [2]. For particle size analysis, clay, silt, and sand contents in the soil sample were determined using the micropipette method [31]. Soil pH and EC were measured in 1:5 DI water:air-dried soil using a pH meter (MP220, Mettler Toledo, Leicester, UK) and an EC meter (MC226, Mettler Toledo, Leicester, UK), respectively. The air-dried soil sample was sieved with a 0.5 mm sieve and digested with concentrated sulfuric acid (H_2_SO_4_) and Kjeltabs Se/3.5^®^ (3.5 g K_2_SO_4_ + 3.5 mg Se) for the analysis of total nitrogen [32]. Then, the T-N concentration was determined using a Kjeldahl analyzer (Kjeldahl 2300, Foss, Hillel, Denmark). Following extraction with a 2 M KCl solution, the soil sample was steam-distilled with magnesium oxide (MgO) powder, and the ammonium concentration was determined using the Kjeldahl analyzer. The sample was steam-stilled again with MgO and Devarda’s alloy powders. Then, the nitrate concentration was determined using a Kjeldahl analyzer. Available phosphorus (Av. P_2_O_5_) was determined colorimetrically using a UV spectrophotometer (UV-160 A, Shimadzu, Kyoto, Japan) at 880 nm following the Bray No. 1 extraction method [33]. Cation exchangeable capacity (CEC) and soluble macronutrients, including Ca, K, and Mg, in the soil samples were determined using a Kjeldahl 2300 distiller and inductively coupled plasma–optical emission spectroscopy (ICP–OES) (Agilent 5110, Agilent Technologies, Santa Clara, CA, USA), respectively, following extraction with 1 M NH_4_OAc solution at pH 7.0 [30].

#### 2.3.3. Emission of CH_4_ and N_2_O

**Figure 1:**
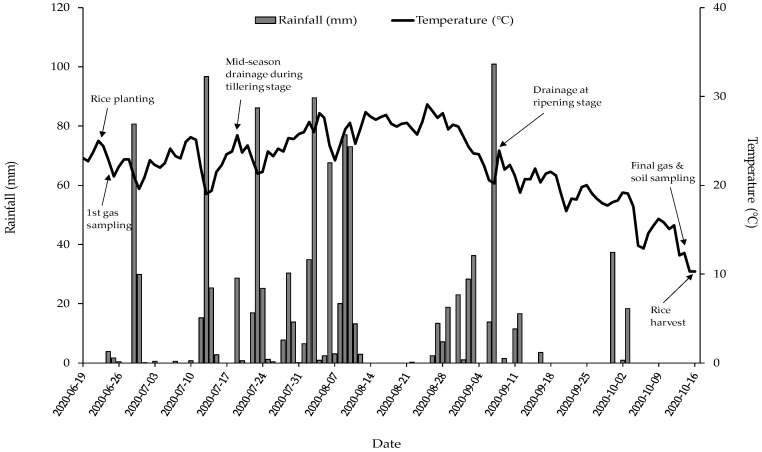
Precipitation and average temperature in the experimental paddy fields measured by the Hongseong weather station of Korea Meteorological Administration.

The portable static-closed chambers (diameter: 32 cm, height: 130 cam) were installed 30 min before gas sampling to collect CH_4_ and N_2_O gases emitted from the paddy fields. Gas measurement was conducted nine times over the rice cultivation period on the 7th, 14th, 26th, 39th, 46th, 56th, 77th, 91st, and 115th day from the rice planting. Climatic information during gas sampling was obtained from the Hongseong weather station of Korea Meteorological Administration (Figure 1). Generally, the increasing trend of daily average temperature during the field experiment was 8 °C at the first gas measurement and 25.5 °C at the second measurement. The gas samplings were performed between 10 a.m. and 3 p.m. daily. The headspace volume was recorded differently depending on the water level, ranging from 82.3 to 98.0 L. The soil surface area was 784 cm^2^. The aliquots (approximately 15 mL) of headspace gas were injected at 0, 15, and 30 min after sealing. The GHG concentrations at each sampling time were determined in situ using the GT5000 Terra portable gas analyzer (Gasmet Technologies, Vantaa, Finland). 

### 2.4. Statistical Analysis

All data indicate the mean of the triple measurements. One-way ANOVA with Tukey’s honestly significant difference post hoc test at the 0.05 probability level was conducted (*n* = 3) to compare differences in SOC fractions and biogeochemical properties among different fertilization practices. Pearson’s correlation analysis was conducted to investigate how soil organic C fractions correlate with soil parameters. Principal component analysis (PCA) was performed focusing on the relationships of different SOC fractions with soil and GHG parameters to identify patterns of variation in the dataset. Additionally, a linear regression test was conducted to investigate the association of SOC fractions with GHG emissions, which may provide a potential for SOC fractions to be used as a GHG emission index. R (version 3.3.3) was used to conduct all statistical analyses in this study.

## 3. Results

### 3.1. SOC Fractions

The concentrations of all types of SOC fractions varied according to the type of fertilization practices (Table 2). Among the fractions, LOIC had the highest concentration in O5 paddy soil (72.7 g kg^−1^), followed by O3, O6, C1, O4, O2, O1, and C1 (45.5 g kg^−1^). The O5 soil treated with CM + RS had a significantly greater LOIC compared to soils of other sites (*p* < 0.05). The O3 soil treated with CM + WS induced an intermediate value significantly higher than O1 and C1 soils treated with OCM + WS and NPK, respectively (*p* < 0.05). The highest concentration (24.8 g kg^−1^) for MBC was observed in the O5 soil. The other RS-treated soils of C2 and O6 sites had higher WBC concentrations (>19.0 g kg^−1^) than the RS-untreated soils. POXC was in the order O5 (853 mg kg^−1^), O6, C2, O2, C1, O3, O4, and O1 (450 mg kg^−1^). Like WBC, the soils of C2, O5, and O6 contained higher POXC than the soils of the remaining sites. The highest PMC concentration was observed in the O6 soil (313 mg kg^−1^), while C2 soil represented the lowest concentration (76.6 mg kg^−1^). The MBC concentrations among the sites were in the following order: O5, O6, O4, C1, C2, O2, O1, and O3. Remarkably, the MBC values in O5 and O6 soils (>133 mg kg^−1^) were approximately three times higher than those observed in the remaining soils (<48 mg kg^−1^). 

### 3.2. Physicochemical Soil Properties

Differences in the soil physicochemical properties of paddy sites are shown in Table 3. There was no significant difference in soil BD among all paddy sites (*p >* 0.05). For soil chemistry, the highest values for each relevant parameter were observed in different treatments: pH (7.13) in C1; EC (0.233 dS m^−1^) in O1; T-N (2.03 g kg^−1^), NH_4_ (140.8 g kg^−1^), CEC (21.6 cmol_c_ kg^−1^), Ex. Ca, K, and Mg (10.3, 1.23, and 3.83 cmol_c_ kg^−1^, respectively) in O5; NO_3_ (8.99 g kg^−1^) in O3; and Av. P_2_O_5_ (106 mg kg^−1^) in O6. Values of soil pH, EC, CEC, and Ex. K in all paddy soils were within or over the optimum range for rice cultivation recommended by the Rural Development Administration, South Korea [34]. On the other hand, C1, C2, O1, O3, O4, and O6 had a lower Av. P_2_O_5_ concentration than the optimum range. At the same time, O2 had lower concentrations of Ex. Ca and Mg. The proportions of clay, silt, and sand in soil particle distribution differed according to the site. The soil texture of all paddy fields was as follows: sandy loam for O2; silt loam for C1, C2, O1, O3, and O6; silt for O5; and silt clay for O6. 

### 3.3. GHG Emission

The cumulative emission of GHGs from paddy fields during the growing season, from rice planting to harvest, varied depending on the sites (Table 4). The cumulative CH_4_ flux from all paddies ranged from 90.8 to 903 kg ha^−1^ d^−1^; the highest value was observed at the O3 soil, while O1 soil represented the lowest value. Soils of O5 and O6 treated with CM + RS induced intermediate values that were significantly higher (approximately 1.8–8.3 times) than C2 (NPK + RS), O1 (OCM + WS), and O4 (CM + WS) soils (*p* < 0.05). For N_2_O emission, the O5 soil had the highest value (0.28 kg ha^−1^). There was no significant difference in the values among the remaining sites, ranging from –3.12 to –1.06 kg ha^−1^ (*p* > 0.05). 

### 3.4. Relationships of SOC Fractions with Soil Prameters and GHGs 

**Figure 2:**
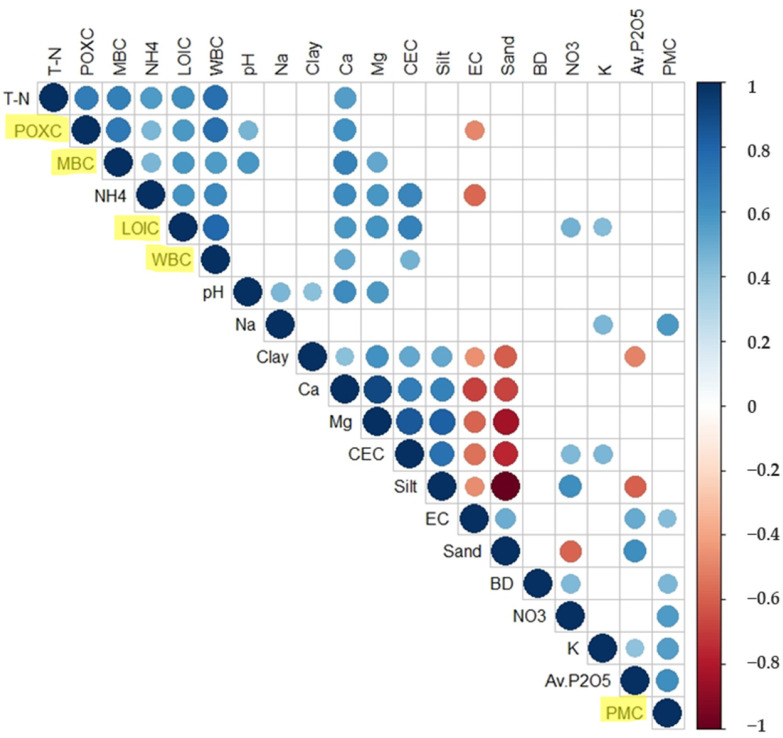
Correlation matrix among soil organic C fractions (LOIC, WBC, POXC, PMC, and MBC; highlighted in yellow) and soil biogeochemical parameters (bulk density; pH; EC; T-N; ammonium; nitrate; CEC; soluble P, Ca, K, and Mg; and particles including clay, silt, and sand). The areas of circles show the value of corresponding Pearson correlation coefficients with significance at the 0.05 probability level. Correlation values are displayed by circle in the upper panel; positive correlations are shown in blue and negative correlations in red. Color intensity (light to dark) and the size of the circle (small to big) are proportional to the correlation coefficients (0 to 1 for positive and 0 to −1 for negative), where, for example, the correlation coefficients on the principal diagonal are equal to 1, represented in dark blue and largest size of circle. The legend on the right side of the correlogram shows the Pearson correlation coefficients with their corresponding colors.

The correlations between SOC fractions and soil properties measured in this study are shown in Figure 2. There were significant positive correlations among the SOC fractions (*p* < 0.05), except for PMC. Among SOC fractions, LOIC was positively correlated with T-N, NH_4_, NO_3_, Ex. Ca, Ex. Mg, Ex. K, and CEC (r = 0.61, 0.59, 0.48, 0.59, 0.60, 0.43, and 0.68, respectively). WBC had significant positive correlations with T-N, NH_4_, CEC, and Ex. Ca (r = 0.75, 0.65, 0.47, and 0.52, respectively). POXC was positively correlated with pH, T-N, NH_4_, and Ex. Ca (r = 0.46, 0.70, 0.45, and 0.61, respectively). At the same time, PMC was positively correlated with NO_3_, Av. P_2_O_5_, and Ex. K (r = 0.57, 0.61, and 0.56, respectively). MBC was significantly correlated with pH, T-N, NH_4_, CEC, and Ex. Ca and Mg (r = 0.58, 0.69, 0.46, 0.67, and 0.52, respectively).

**Figure 3:**
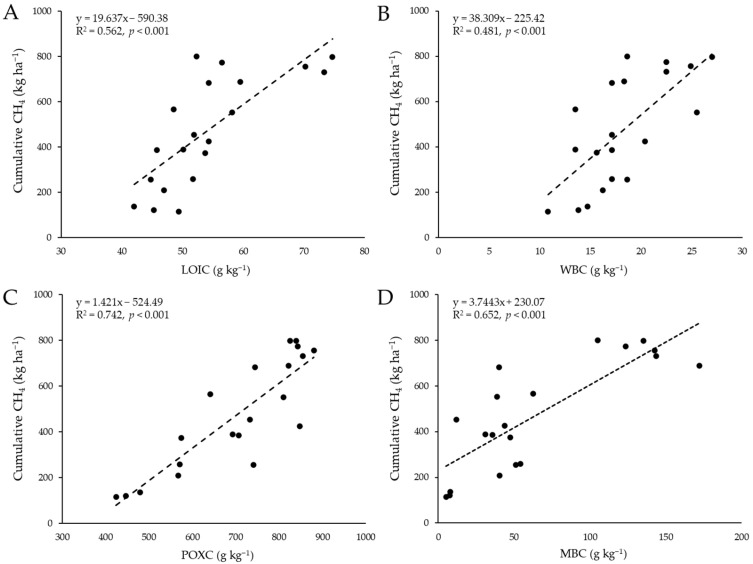
Linear regression relationship of cumulative methane (CH_4_) emission with different SOC fractions, including LOIC (**A**), WBC (**B**), POXC (**C**), and MBC (**D**).

According to the linear regression analysis (Figure 3), SOC fractions tended to have significant relationships with the cumulative emission of GHGs, particularly methane. The strongest linear relationship of cumulative CH_4_ emission was observed with POXC (R^2^ = 0.74), followed by MBC, LOIC, and WBC (R^2^ = 0.65, 0.56, and 0.48, respectively). On the other hand, PMC did not have a significant relationship with CH_4_ emission (R^2^ = 0.08, *p* > 0.05).

### 3.5. Interpretation of PCA

**Figure 4:**
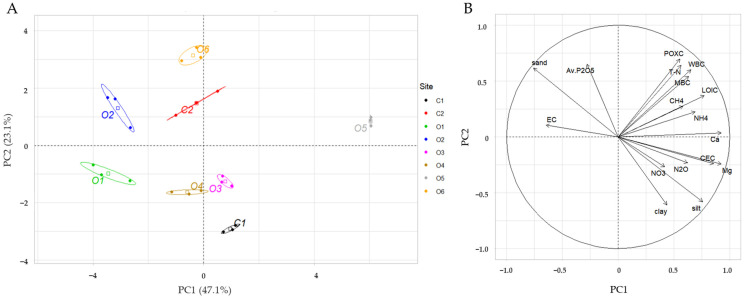
PCA of the biogeochemical properties of paddy soils with different fertilization practices. (**A**) Component scores for first two principal components of eight different paddy fields. (**B**) Loading plot of soil carbon fractions and biogeochemical parameters.

A clear separation of paddy fields managed with different long-term fertilization practices was provided by the PCA result of the dataset for all soil and gas variables (Figure 4). Along the PC1 axis (47.1% variance), paddy fields with soil management types were split primarily due to SOC fractions, such as LOIC, MBC, WBC, and POXC, as well as EC, Ex. Mg and Ca, CEC, ammonium, T-N, CH_4_, N_2_O, sand, and silt (*p* < 0.01, Table S2). Along the PC2 axis (23.1% variance), the paddies were separated by Av. P_2_O_5_ (*p* < 0.001, Table S2). The overall trend of the multivariate analysis showed that application of integrated organic amendments, such as CM + RS, had strong influence on most SOC fractions (LOIC, WBC, POXC, and MBC), major soil variables (soluble N, P, Ca, etc.), GHG emissions, and their significant correlations.

## 4. Discussion

Higher organic matter content in cultivated soil has more beneficial effects on overall soil biogeochemical processes, which contribute to sustainable crop production systems. However, despite this widely known fact, the SOM status of agricultural lands has degraded globally due to various anthropogenic activities (e.g., overuse of chemicals and intensive tillage) and environmental changes (e.g., climate, fire, and soil erosion), thus affecting the quality and productivity of agricultural soils [35]. Hence, many farmers have strived for a continuous input of organic additives taken from elsewhere, including crop residues, animal manures, biowastes, etc., to rehabilitate the declined SOC stock on agricultural lands. Nonetheless, it has often not been as effective as intended for sustainable agricultural production. This is due to the beneficial effects of SOA added, i.e., C sequestration and soil function improvement, which could vary considerably with the amount and quality of SOA as well as diverse external factors, such as soil characteristics, climate, land management practices (e.g., fertilization, tillage, and cover cropping), and the cropping system (e.g., crop species, irrigation, and cultivation type) [36,37,38]. Moreover, soil management practices based on the quantity of total SOC content measured by LOI and WB methods have been frequently proven ineffective [11,21]. Therefore, this calls for alternative methods through precise and steady monitoring of various types of SOC fractions highly sensitive to spatiotemporal change in agriculture systems. 

In this study, we confirmed that the formation of SOC fractions in paddy fields varied significantly with different long-term fertilization practices, as shown in Table 2. The paddy soils applied with organic fertilizers alone, i.e., the integration of CM + RS, CM + WS, and OCM + WS, and combined with NPK fertilizer, i.e., NPK + RS, showed better improvement in SOC fractions, except for MBC, than paddy soil applied with chemical fertilizer alone, indicating that all types of SOA added played a progressive role in forming the well-balanced SOC pool structure. Remarkably, the greatest values of all SOC fractions in two organic paddy fields, such as O5 and O6, may suggest that the combined application of cow manure and RS as basal fertilizers could be the best soil management practice. Such a practice would lead to the most balanced improvement in the overall SOC pool of this paddy field area, beyond the influence of other organic combinations. Cow manure, an agricultural waste, is widely used as an organic fertilizer source to primarily increase the SOM stock of paddy lands in South Korea. This livestock manure has high efficiency in SOM mineralization and can improve the availability of C, microbial abundance and activity [39,40], and soil nutrient status of N and P [25,41]. Therefore, its application can contribute to sustaining crop yield and soil health. Yan et al. [42] demonstrated that the long-term application of manure in paddy soil could improve labile SOC fractions more than NPK application. A similar phenomenon was observed in this study, with significant differences observed in concentrations of POXC and PMC between paddy soils of NPK + RS and CM + RS (*p* < 0.05). Additionally, soil management via the application of RS back to paddy fields has been widely practiced to ameliorate the SOC stock and improve soil biogeochemistry and fertility. This practice can contribute to an increase in crop yield and sustainability of agroecosystem services [41,43,44,45,46]. According to Liu et al. [44], SOC stock in flooded paddies increased by approximately 20 Mg ha^−1^ for 20 years by NPK + RS application. This corresponds to an overall SOC deposition rate (1.0 Mg ha^−1^ yr^−1^) that is double of NPK application alone (0.48 Mg ha^−1^ yr^−1^).

Similarly, through a two-year field experiment, Lee et al. [47] reported that NPK + RS application in paddy fields increased the SOC stock at the rate of 1.5 to 2.8 Mg ha^−1^ yr^−1^. In contrast, a negative rate of 0.24 to 1.1 Mg ha^−1^ yr^−1^ was observed in paddies with NPK application alone. In this study, we confirmed a clear increase in the SOC pool by the effect of RS return to the rice fields by comparing the degree of SOC fractions between two paddy fields (C1 vs. C2 sites) of long-term conventional farming practice (Table 2), namely, the significantly higher LOIC, WBC, POXC, and PMC concentrations (15, 63, 21, and 178%, respectively) in C2 soil treated with NPK + RS than in C1 soil treated with NPK alone. This result may indicate that long-term RS return as a basal fertilizer source could improve the overall SOC pool of rice cultivation lands. In addition, it seems that the long-term application of crop residues, such as RS, into the soil could significantly affect the labile SOC pool rather than the total SOC stock [36]. Consistent with our finding, Wang et al. [45] reported that additional RS application increased total SOC by 2% but more labile SOC fractions, including DOC, POXC, and MBC, by 10–23%. 

As is well known, improving SOC pool by addition of SOA can boost soil fertility by enhancing C utilization efficiency for soil microorganisms that promote SOA-derived nutrient mineralization [2,11,12]. Consistent with this, we found significant correlations of SOC fractions with soil chemical variables related to soil nutrition in this study (see Figure 2). However, the effect on soil chemical parameters sometimes differed among the same basal fertilizer treatment group. For example, like SOC fractions, total N content in both O5 and O6 paddy soils of CM + RS treatment was highest simultaneously, while concentrations of CEC and soluble nutrients, including NH_4_, NO_3_, and extractable Ca, K, and Mg, were highest in O5 soil but exceptionally low in O6 soil. Such variations in the soil nutrient parameters between the two paddies may be attributed to differences in soil particle size distribution [48], topography [49], and land management practices [50]. Our results showed that O5 soil containing higher clay content compared to O6 soil (Table 3) had higher concentrations of SOC fractions and soluble nutrients, similar to the results reported by Wang et al. [48] and Arunrat et al. [49]. Additionally, we found significant positive correlations of SOC fractions and soluble nutrients with silt content but negative correlations with sand content (Figure 2). This suggests that specific soil particles could be crucial in regulating the overall SOC pool and agricultural soil fertility [49]. Furthermore, it might be considered that differences in the quality, flow direction, and drainage frequency of paddy irrigation water and the rice variety among the paddies induced these differences in this field study area. 

Nevertheless, the O5 and O6 sites consistently had approximately three times higher MBC concentrations in soils than the other sites. This result suggests that the long-term application of CM + RS as a basal fertilizer is the most effective soil management practice in terms of facilitating the soil microbial community. Thus, it would aid in improving the soil health and sustainability of rice production in this arable paddy area [22,51]. Microbial biomass is highly associated with the C cycling process in agricultural soil, and its pool size relies on the degree of labile SOC fractions [42]. This could be verified in this study through the fact that MBC concentrations not only had the greatest correlation coefficient with POXC (r = 0.72, *p* < 0.001) but also had a robust linear regression with cumulative CH_4_ emission (R^2^ = 65%, *p* < 0.001). Likewise, Kumar et al. [40] demonstrated that increased labile SOC through the long-term application of chemical and organic fertilizers allowed promotion of the soil microbial community, especially copiotrophic bacteria, thereby improving the soil health of the farmland. Moreover, high correlations of MBC with NH_4_ and extractable Ca and Mg (Figure 2) indicated that soil microbial biomass is likely to play a crucial role in the dynamics of other nutrients in belowground rice fields, consequently affecting rice yield. 

Flooded paddy soil is a significant source of GHGs, such as methane and nitrous oxide, which greatly influence global warming. CH_4_ and N_2_O gases are currently reported to have high global warming potential, i.e., 27 and 273 times that of CO_2_ at a 100-year time horizon, respectively [52]. Therefore, they are considered as key contributors to global climate change. Applying organic amendments and inorganic fertilizers in paddy fields can increase GHG emissions but vary with external factors, such as climate, soil properties, rice varieties, and land management practices [26]. In addition, both GHGs may represent different responses to prolonged submergence conditions of the rice fields. Adding organic materials to waterlogged rice fields increases CH_4_ emission. At the same time, the constant submergence condition may generate low N_2_O emissions by stimulating the reduction of soil nitrate and nitrite to N_2_ gas in rice fields [26]. Meanwhile, additional N fertilizer input has been reported to significantly increase nitrous oxide emissions [53,54]. In this study, the methane flux appeared to fluctuate broadly by mid-water drainages during rice cultivation. The N_2_O flux was rapidly generated after topdressing fertilization by adding a nitrogen source (Figure S2). Among the rice fields, the O5 paddy site treated with CM + RS seemed to release more significant amounts of CH_4_ and N_2_O from the soil. This may be attributed to the high availability of C and N in the soil compared to other sites (see Table 2 and Table 3). Notably, the most significant linear relationship between cumulative CH_4_ emission and soil POXC concentration (R^2^ = 74%, *p* < 0.001) could prove this statement (Figure 3). Regarding cumulative N_2_O, it had a significant linear relationship with PMC concentration (R^2^ = 36%, *p* < 0.001) but not with POXC concentration (R^2^ = 1.1%, *p* > 0.05). This indicates that the presence of more readily mineralizable SOM in the rice fields has a high potential to contain more N sources capable of gradually being denitrified by soil microorganisms, such as denitrifying bacteria [55]. 

Considering that this study was conducted through descriptive field research, it was not enough to determine the direct impact of the treated fertilizer sources on SOC pool structure, soil fertility, microbial biomass, GHG emissions, etc. Although the type and amount of fertilizer sources treated in all rice fields for an extended period were constant, the existence of differences in the timings of fertilizer application, irrigation, and mid-water drainage; conditions of irrigation water; and type of land cover management (i.e., tillage and cover crops) appeared to produce rather unexpected results. Therefore, it could be thought that these external differences sometimes resulted in data variations between sites treated with different fertilizer sources and even between the same group of basal fertilizer treatments. Despite this large variability in the data, based on all our findings, the long-term fertilization of CM + RS across all paddy fields could be the most efficient paddy management method in this rice-producing region. Moreover, the strong relationship of SOC fractions, especially POXC, with soil nutrient status and microbial abundance suggests that the labile C fraction could efficiently estimate soil fertility and health for sustainable rice production, further predicting soil GHG emissions. 

## 5. Conclusions

Paddy soils have a lower SOC content than natural lands, so they can have a high potential for C storage through SOA input and a combination of suitable land management practices, such as less tillage, crop residue return, drainage control, etc. Our findings from this one-year descriptive field study on different long-term fertilization practices for rice cultivation revealed that SOC stock and its fractions varied significantly with the basal fertilizer sources. Application of the integrated amendment of CM + RS tended to generate the best efficacy in increasing both recalcitrant and labile C pools and plant availability of nutrients (e.g., NH_4_, NO_3_, Av. P_2_O_5_, Ex. K, etc.), thus contributing to improving soil quality and fertility. Meanwhile, paddy fields treated with CM + RS emitted the largest amount of methane. The emitted CH_4_ showed significant relationships with POXC and MBC across the fields. This could suggest that the enhanced formation of labile C and soil microbial community affected the C cycle, leading to increased GHG release from the agricultural lands. Overall, the incorporated application of cow manure and RS into paddy fields seems to be the best fertilization practice in this rice cultivation area. Using this method helps to improve the balance of SOC pool structure and soil biogeochemical properties, which are significant factors in enhancing the sustainability of agroecosystem services. It is further necessary to maximize these benefits (e.g., improvement of soil quality and fertility) and minimize the disadvantages (e.g., GHG release and C loss) by developing more suitable fertilizers and soil management approaches for paddy fields. In this way, it will be possible to achieve sustainable agricultural production more effectively. 

## Figures and Tables

**Table 1:** Description of eight paddy fields used in this study according to the land management practices.

Site	Area (ha)	GPS Coordinates	Type	Basal Fertilizer	Topdressing Fertilizer
C1	1.155	36°32′47″ N 126°41′55″ E	Conventional	NPK	NK
C2	1.504	36°32′59″ N 126°42′07″ E	Conventional	NPK + RS	NK
O1	1.480	36°33′02″ N 126°42′02″ E	Organic	OCM + WS	MC
O2	2.010	36°32′44″ N 126°41′39″ E	Organic	OCM + WS	MC
O3	2.990	36°32′45″ N 126°41′54″ E	Organic	CM + WS	MC
O4	2.110	36°32′46″ N 126°41′48″ E	Organic	CM + WS	MC
O5	2.810	36°32′44″ N 126°41′45″ E	Organic	CM + RS	MC
O6	3.040	36°32′39″ N 126°41′50″ E	Organic	CM + RS	MC

NPK (63:30:21 = N:P_2_O_5_:K_2_O; kg ha^−1^); CM (cow manure, 30 t ha^−1^); OCM (oilseed cake manure; 2 t ha^−1^); RS (rice straw, 100% turnover); WS (wheat straw, 100% turnover); NK (63:9 = N:K_2_O, kg ha^−1^); MC (manure compost containing 9% of nitrogen, 200 kg ha^−1^).

**Table 2:** Concentrations of soil organic carbon fractions, such as LOIC, WBC, POXC, PMC, and MBC, in paddy soils treated with different fertilization practices.

Site	LOIC	WBC	POXC	PMC	MBC
	(g kg^−1^)		(mg kg^−1^)		
C1	45.5 c	13.2 c	663 cd	213 c	45.6 b
C2	52.4 bc	21.5 ab	799 ab	76.6 d	44.6 b
O1	45.6 c	13.1 c	450 f	236 bc	6.90 b
O2	50.7 bc	17.1 bc	728 bc	265 ab	29.2 b
O3	60.0 b	18.9 b	630 de	243 bc	6.40 b
O4	50.8 bc	16.3 bc	571 e	277 ab	47.3 b
O5	72.7 a	24.8 a	853 a	269 ab	141 a
O6	56.1 bc	19.8 ab	835 a	313 a	133 a

Data are the mean of three replicates. Same letters in each column indicate no significant difference among the treatments (Tukey’s HSD post hoc test, *p* < 0.05). LOIC, loss-on-ignition carbon; WBC, Walkley–Black carbon; POXC, permanganate oxidizable carbon; PMC, potentially mineralizable carbon; MBC, microbial biomass carbon.

**Table 3:** Physicochemical properties of paddy soils treated with different fertilization practices.

Site	Bulk Density	pH_1:5w_	EC	T-N	NH_4_	NO_3_	Av. P_2_O_5_	CEC	Ex. Ca	Ex. K	Ex. Mg	Clay	Silt	Sand
			(dS m^−1^)	(g kg^−1^)	(mg kg^−1^)	(g kg^−1^)	(mg kg^−1^)	(cmol_c_ kg^−1^)	(cmol_c_ kg^−1^)	(cmol_c_ kg^−1^)	(cmol_c_ kg^−1^)	(%)	(%)	(%)
C1	1.53 a	7.13 a	0.100 b	1.10 c	23.8 cd	3.09 e	16.4 d	15.1 b	8.63 b	0.43 d	3.01 b	7.37 a	78.2 b	14.4 f
C2	1.57 a	6.27 d	0.100 b	1.93 a	82.8 b	0.878 f	24.6 d	12.6 b	7.13 d	0.30 e	1.68 f	3.07 bc	58.1 d	38.9 c
O1	1.73 a	6.20 d	0.233 a	1.20 bc	38.4 c	3.13 e	64.8 b	11.5 b	5.03 g	0.83 b	1.52 g	2.83 bc	57.4 d	39.7 c
O2	1.63 a	6.53 c	0.200 a	1.13 bc	17.4 cd	2.57 e	94.5 a	11.5 b	4.80 h	0.93 b	1.47 h	4.47 abc	40.2 f	55.3 a
O3	1.73 a	6.47 c	0.200 a	1.50 b	13.7 cd	8.99 a	35.4 cd	15.2 b	6.80 e	0.60 c	2.35 c	4.77 abc	76.7 bc	18.6 e
O4	1.73 a	6.50 c	0.167 ab	1.33 bc	13.4 cd	8.05 b	54.9 bc	14.2 b	5.73 f	0.90 b	2.00 d	4.43 abc	74.0 c	21.6 d
O5	1.73 a	6.80 b	0.100 b	2.03 a	140.8 a	6.69 c	60.8 bc	21.6 a	10.3 a	1.23 a	3.83 a	5.70 ab	82.9 a	11.4 f
O6	1.73 a	7.00 a	0.200 a	1.97 a	11.0 d	4.81 d	106 a	10.4 b	7.43 c	0.53 cd	1.90 e	1.97 c	53.6 e	44.5 b
Optimum range ^†^	–	5.5–6.5	≤2.0	–	–	–	80–120	10–15	5.0–6.0	0.25–0.30	1.5–2.0	–	–	–

Data are the mean of three replicates. Same letters in each column indicate no significant difference among the treatments (Tukey’s HSD post hoc test, *p* < 0.05). EC, electrical conductivity; T-N, total nitrogen; NH_4_, ammonium; NO_3_, nitrate; Av. P_2_O_5_, available phosphorus; CEC, cation exchangeable capacity; Ex. Ca, exchangeable calcium; Ex. K, exchangeable potassium; Ex. Mg, exchangeable magnesium. ^†^ Recommended values for rice cultivation [34].

**Table 4:** Cumulative fluxes of methane (CH_4_) and nitrous oxide (N_2_O) emitted from paddy soils treated with different fertilization practices during the growing season.

Site	CH_4_	N_2_O
	(kg ha^−1^)	(kg ha^−1^)
C1	541 b	−1.23 ab
C2	411 bc	−3.49 c
O1	91 d	−2.01 bc
O2	507 b	−3.12 c
O3	903 a	−1.69 bc
O4	280 cd	−1.06 ab
O5	762 a	0.28 a
O6	754 a	−1.65 bc

Data are the mean of three replicates. Same letters in each column indicate no significant difference among the paddy sites (Tukey’s HSD post hoc test, *p* < 0.05).

## Data Availability

Not applicable.

## Supplementary Materials

The following supporting information can be downloaded at: https://www.mdpi.com/article/10.3390/plants11233195/s1, Table S1: Properties of soil organic amendments applied to paddy fields; Table S2: Loadings of variables evaluated through PCA in this study; Figure S1: Location of rice fields where different fertilizer practices have been carried out; Figure S2: The CH_4_ and N_2_O flux emissions from paddy soils treated with different long-term fertilizer treatments during the rice growth period: C1 (NPK), C2 (NPK + RS), O1 and O2 (OCM + WS), O3 and O4 (CM + WS), and O5 and O6 (CM + RS). RS, rice straw; OCM, oilseed cake manure; WS, wheat straw; CM, cow manure.
