# Supplementary material for: Co-Responses of Soil Organic Carbon Pool and Biogeochemistry to Different Long-Term Fertilization Practices in Paddy Fields
Source: Plants (Basel). 2022 Nov 22;11(23):3195. doi: 10.3390/plants11233195 (PMC9737529; doi:10.3390/plants11233195)
Supplement: Supplementary file 1 [file plants-11-03195-s001.zip › plants-1983416-supplementary.pdf]

Supplementary Materials

Table S1. Properties of soil organic amendments applied to paddy fields.

Type	N	P	K	C
	-- (%)--			
CM	0.40	0.24	0.07	23
OCM	4.5	0.65	0.83	41
RS	0.72	0.14	1.4	48
WS	0.70	0.14	1.2	50
MC	9.0	0.44	1.7	35

CM, cow manure; OCM, Oilseed cake manure; RS, rice straw; WS, wheat straw; MC, manure compost

Table S2. Loadings of variables evaluated through PCA in this study.

Soil parameter	PC1	PC2
LOIC	0.771	0.370
WBC	0.651	0.601
POXC	0.553	0.696
MBC	0.630	0.544
EC	-0.645	0.104
T-N	0.563	0.640
NH_4	0.688	0.225
NO_3	0.415	-0.269
Av. P_2O_5	-0.281	0.651
CEC	0.856	-0.242
Ca	0.923	0.035
Mg	0.924	-0.244
Clay	0.437	-0.609
Sand	-0.757	0.616
Silt	0.756	-0.581
CH_4	0.579	0.273
N_2O	0.622	-0.232

LOIC, loss-on-ignition carbon; WBC, Walkley-Black carbon; POXC, permanganate oxidizable carbon; MBC, microbial biomass carbon; EC, electrical conductivity; T-N, total nitrogen; NH_4, ammonium; NO_3, nitrate; Av. P_2O_5, available phosphorus; CEC, cation exchangeable capacity; Ex. Ca and Mg, exchangeable calcium and magnesium.

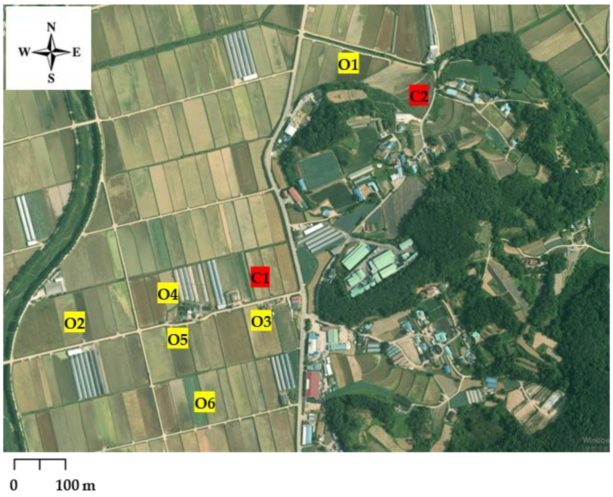

Site	Basal fertilizer	Topdressing fertilizer
C1	NPK	NK
C2	NPK + Rice straw	
O1 & O2	Oilseed cake manure + Wheat straw	Manure compost
O3 & O4	Cow manure + Wheat straw	
O5 & O6	Cow manure + Rice straw	

Figure S1. Location of rice fields where different fertilizer practices have been carried out.

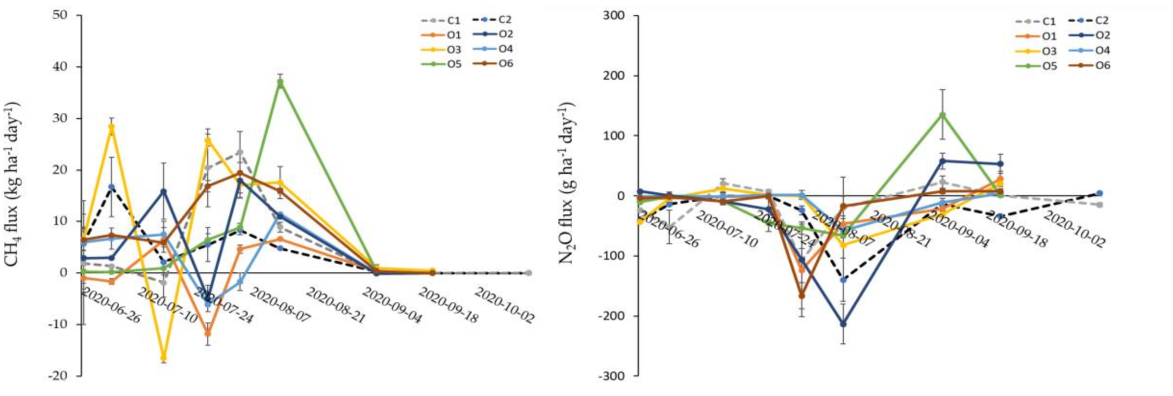

Figure S2. The CH_4 and N_2O flux emissions from paddy soils treated with different long-term fertilizer treatments during the rice growth period: C1 (NPK), C2 (NPK + RS), O1 and O2 (OCM + WS), O3 and O4 (CM + WS), and O5 and O6 (CM + RS). RS, rice straw; OCM, oilseed cake manure; WS, wheat straw; CM, cow manure.
